# Conjugation of amiodarone to a novel cardiomyocyte cell penetrating peptide for potential targeted delivery to the heart

**DOI:** 10.3389/fchem.2023.1220573

**Published:** 2023-07-12

**Authors:** Ray Yurko, Kazi Islam, Beth Weber, Guy Salama, Maliha Zahid

**Affiliations:** ^1^ Peptide Synthesis Facility, University of Pittsburgh, Pittsburgh, PA, United States; ^2^ Division of Cardiology, Department of Medicine, Pittsburgh Heart, Lung, Blood, and Vascular Medicine Institute, University of Pittsburgh School of Medicine, University of Pittsburgh Medical Center, Pittsburgh, PA, United States; ^3^ Deptartment of Cardiovascular Diseases, Mayo Clinic, Rochester, MN, United States

**Keywords:** cardiac targeting peptide, cardiomyocytes, amiodarone hydrochloride, cell penetrating peptides, protein transduction domain, cardiac targeting peptide, amiodarone

## Abstract

Modern medicine has developed a myriad of therapeutic drugs against a wide range of human diseases leading to increased life expectancy and better quality of life for millions of people. Despite the undeniable benefit of medical advancements in pharmaceutical technology, many of the most effective drugs currently in use have serious limitations such as off target side effects resulting in systemic toxicity. New generations of specialized drug constructs will enhance targeted therapeutic efficacy of existing and new drugs leading to safer and more effective treatment options for a variety of human ailments. As one of the most efficient drugs known for the treatment of cardiac arrhythmia, Amiodarone presents the same conundrum of serious systemic side effects associated with long term treatment. In this article we present the synthesis of a next-generation prodrug construct of amiodarone for the purpose of advanced targeting of cardiac arrhythmias by delivering the drug to cardiomyocytes using a novel cardiac targeting peptide, a cardiomyocyte-specific cell penetrating peptide. Our *in vivo* studies in guinea pigs indicate that cardiac targeting peptide-amiodarone conjugate is able to have similar effects on calcium handling as amiodarone at 1/15th the total molar dose of amiodarone. Further studies are warranted in animal models of atrial fibrillation to show efficacy of this conjugate.

## Introduction

Atrial fibrillation is the most common rhythm disturbance in clinical practice affecting the heart, with an estimated prevalence of 10% in those over 80 years of age (Staerk et al., 2018). It is estimated that 3 to 6 million US adults suffer from it, with prevalence expected to double by year 2040. Although, not immediately life threatening, it can lead to clot formation in the upper, left chamber of the heart, which upon embolization can cause a stroke. In fact, ∼25% of all strokes are due to atrial fibrillation (Furie, 2020).

**FIGURE 1:**
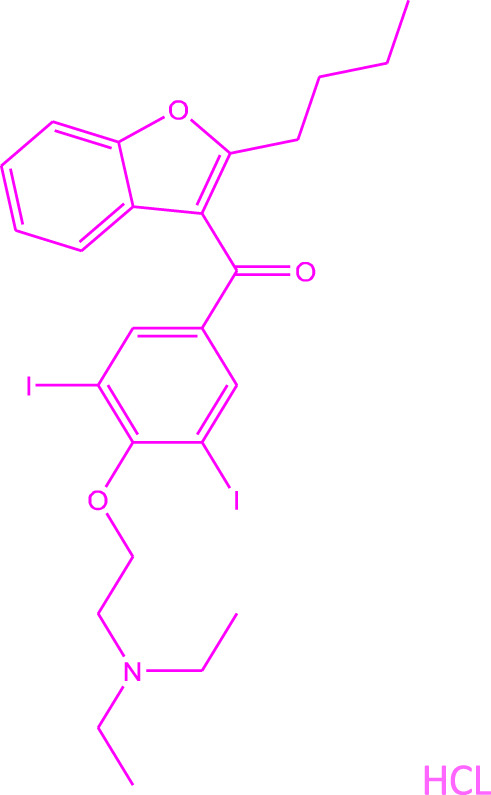
Structure of amiodarone hydrochloride.

The most effective drug shown to keep hearts out of atrial fibrillation and in normal sinus rhythm is Amiodarone hydrochloride (Figure 1). Several clinical trials have demonstrated its superior efficacy over other anti-arrhythmics like Sotalol, Propafenone or Dronedarone (Lumer et al., 2002; Piccini et al., 2009; Singh et al., 2005). Yet, it is a second-line drug for atrial fibrillation, except in patients with congestive heart failure due to reduced life expectancy in that special cohort of patients (January et al., 2019). The reason underlying this choice is the myriad side effects long-term therapy with Amiodarone carries (Mujovic et al., 2020). Although safe enough in the short term to be initiated as outpatient therapy, long term use is associated with significant lung, liver, skin, thyroid and ocular toxicities, limiting use in younger patients with a longer life expectancy.

Cardiac therapeutics, like amiodarone, have long suffered from lack of targeted delivery leading to high doses being administered in order to achieve adequate cardiac levels and efficacy. However, this also leads to uptake by organs other than the heart leading to off-target toxicities. A potential solution would be cell penetrating peptides (CPP) which are short, 5 to 30 amino acid long peptides, with the ability to cross cell membrane barriers while carrying cargoes several times their size in an intact, functional form (Taylor and Zahid, 2020). As so often happens in science, this ability to cross cell membranes by proteins was a serendipitous discovery by two independent groups of scientists working on the HIV coat protein, trans-activator of transcription (Tat) (Green and Loewenstein, 1988; Frankel et al., 1988). Work into the part of this 86 amino acid long protein responsible for transduction led to the identification of an 11 amino acid, arginine/lysine rich residue, Tat, the first CPP (Green et al., 1989). Further work showed its ability to carry a much larger protein fused to it, β-galactosidase, across cell membrane barriers into all tissues after an intra-peritoneal injection and showed its ability to even cross the blood-brain barrier (Schwarze et al., 1999). This robust ability to transduce almost all tissue types also hindered its ability to function as a clinically viable vector.

Our prior work utilizing a combinatorial *in vitro* and *in vivo* phage display approach (Zahid and Robbins, 2011) identified a unique, synthetic, non-naturally occurring peptide, which we termed Cardiac Targeting Peptide (CTP-APWHLSSQYSRT), due to its ability to transduce healthy cardiomyocytes after an intravenous injection (Zahid et al., 2010). Further work into its bio distribution revealed that peak uptake occurs in as little as 15 min with almost complete disappearance of the fluorescently tagged CTP by 6 h (Zahid et al., 2018). The use of CTP as a novel vector targeting cardiomyocytes has been validated by several, independent investigators (Avula et al., 2015; Kim et al., 2021; Gallicano et al., 2022).

The ability of small peptide-based prodrugs, also known as peptide-drug conjugates (PDCs) (Cooper et al., 2021), to specifically target various cancers has been well established as a viable method for delivery of anticancer compounds such Doxorubicin (Nasrolahi Shirazi et al., 2013), Paclitaxel (Ayalew et al., 2017), Pt (IV) (Li et al., 2018) and Monomethyl auristatin E (MMAE) (Cooper et al., 2021) to name a few. In addition, peptide antibiotic prodrugs as next-generation agents effective against resistant microbial infections (Pereira et al., 2015) and mammalian cell targeting siRNA peptide prodrugs have been established as potential therapeutics (Muratovska and Eccles, 2004) as well. In a similar manner, the development of several new technologies specifically designed for the enhanced delivery of Amiodarone to the heart have been developed in recent years. For example, Amiodarone loaded cyclodextrin nanoparticles (Ahmed et al., 2019), Amiodarone containing Poly (lactic-co-glycolic acid (PLGA) nanoparticles (Motawea et al., 2021), and Amiodarone encapsulated hydrogels (Wang et al., 2016) are just a few of the new technologies that have been developed. Despite these technological achievements, to our knowledge, there has never been a documented synthesis of amiodarone covalently conjugated to a CPP like cardiac targeting peptide as a prodrug for the sustained long-term treatment of cardiac arrhythmias.

## Results

### Synthesis of CTP-Thiol

**SCHEME 1:**
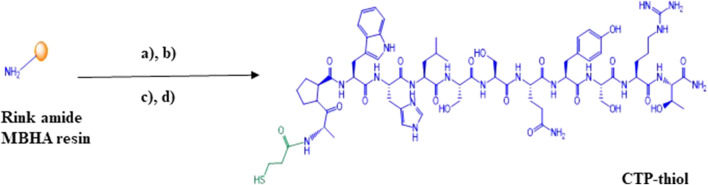
Solid phase synthesis of thiolated cardiac targeting peptide (CTP-thiol). **(A)** (i) Solid. Phase peptide synthesis (Polystyrene solid support of Rink amide MBHA resin is represented by the golden ball, (ii) 20% Piperidine, DMF; **(B)** 3-(Tritylthio)propionic acid, TBTU, HOBt, DIPEA, DMF; **(C)** TFA:TIPS:H_2_O:EDT (94:1:2.5:2.5); **(D)** Preparative HPLC purification followed by MALDI characterization.

Solid phase peptide synthesis of CTP was accomplished on a microwave synthesizer using standard Fmoc/tBu chemistry and Oxyma pure coupling protocols at a 1 mmole scale on Rink amide MBHA resin (10 reactors at 0.1mmole each). After completion of the CTP-peptide chain assembly, all reactor products were pooled and the N-terminal amino group was manually conjugated on-resin with 3-(Tritylthio) propionic acid (5eq.) using TBTU (5eq.)/HOBt (5eq.)/DIPEA (10eq.) in DMF. CTP-thiol was then cleaved from the resin along with side chain deprotection using TFA:TIPS:H_2_O:EDT (94:1:2.5:2.5) for 2 h at room temperature. The resultant crude CTP-thiol product 1 (Scheme 1) was then precipitated in ice cold Diethyl Ether (Et2O) and centrifuged to a pellet @ 2,500 rpm for 5 min. This process was repeated 2 additional times and then the pellet was allowed to air dry in a chemical fume hood for approximately 30 min to remove residual ether from the sample.

Crude CTP-thiol 1 was then dissolved in 50% TFE/0.1%TFA (aq.) in 20 mg batches and then purified by preparative C-18 RP-HPLC. All fractions representing the desired product were pooled and shell frozen in dry ice/ethanol and then lyophilized to a dry powder using an SP Scientific SP VirTis BenchTop Pro freeze dryer. Final yield of the purified CTP-thiol was approximately 60% based on initial reactor synthesis scale and determination of purity was accomplished using analytical C-4 RP-HPLC (Supplementary Figure S1). MALDI analysis was performed to confirm identity of the CTP-thiol product @ m/z 1519.57 [M + H]; 1520.68 (calculated) (Supplementary Figure S2).

### 5-Nitro-2-Pyridinesulfenyl (NPys)-S-CTP synthesis

**SCHEME 2:**
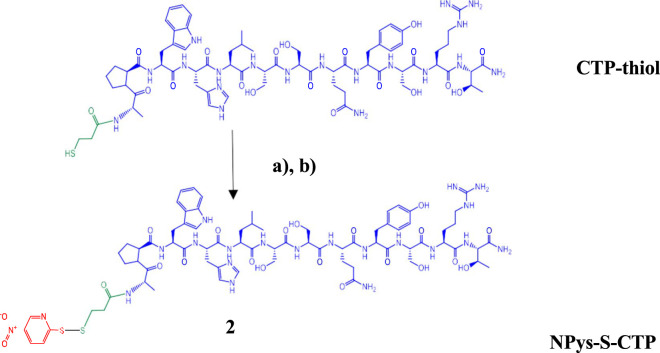
Activation of cardiac targeting peptide. **(A)** 2,2′-Dithiobis (5-nitropyridine) (DTNP) in 80% TFA (aq.) for 15 min; **(B)** Preparative HPLC purification followed by MALDI analysis.

Purified CTP-thiol intermediate 1 was then modified with 2,2′-Dithiobis (5-nitropyridine) (DTNP) in 80% Trifluoroacetic acid (TFA) (aq.) for 15 min at room temperature with gentle mixing (Scheme 2). Samples were then dried to a film using a stream of nitrogen under mild heating conditions. The NPys-S-CTP intermediate 2 film was dissolved in 50%TFE/0.1% TFA and directly purified by preparative C-18 RP-HPLC on a Waters Delta Prep 4,000 chromatography system followed by lyophilization to a dry powder. Determination of final purity was accomplished using analytical C-4 RP-HPLC on a Waters Alliance chromatography system and processed with Empower software (Supplementary Figure S3). The conversion of CTP-Thiol 1 to NPys-S-CTP 2 was nearly quantitative under the reaction conditions applied leading to >90% recoveries. MALDI analysis was performed to confirm the correct mass of NPys-S-CTP at m/z 1674.62 [M + H]; 1674.84 (calculated) (Supplementary Figure S4).

### Amiodarone-thiol synthesis

**SCHEME 3:**
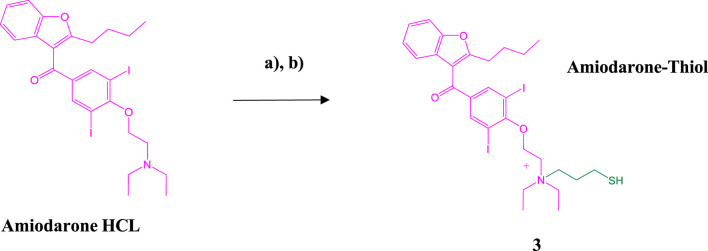
Synthesis of the Amiodarone-thiol intermediate. **(A)** Amiodarone HCl, Sodium iodide (NaI), 1,2,2,6,6-Pentamethylpiperidine, acetonitrile overnight at 37°C; **(B)** Preparative HPLC purification followed by MALDI analysis.

Amiodarone hydrochloride was chloroalkylated at the tertiary nitrogen with a 10% solution of 3- Chloro-1-propanethiol in acetonitrile and a catalytic amount of Sodium iodide and 1,2,2,6,6- Pentamethylpiperidine overnight with gentle mixing at 37°C forming the thiolated quaternary ammonium intermediate 3 (Scheme 3). The crude reaction mix was dissolved in 50% TFE/0.1%TFA and then directly purified by preparative C-18 RP-HPLC followed by lyophilization to a clear film. Determination of purity was accomplished using analytical C-18 RP-HPLC on a Waters Alliance chromatography system along with Empower software (Supplementary Figure S5). MALDI analysis was performed to confirm identity of the amiodarone-thiol product @ 744.8 [M + Na]+; (742.5 calculated for [M + Na]+) (Supplementary Figure S6)

### CTP-amiodarone conjugate synthesis

**SCHEME 4:**
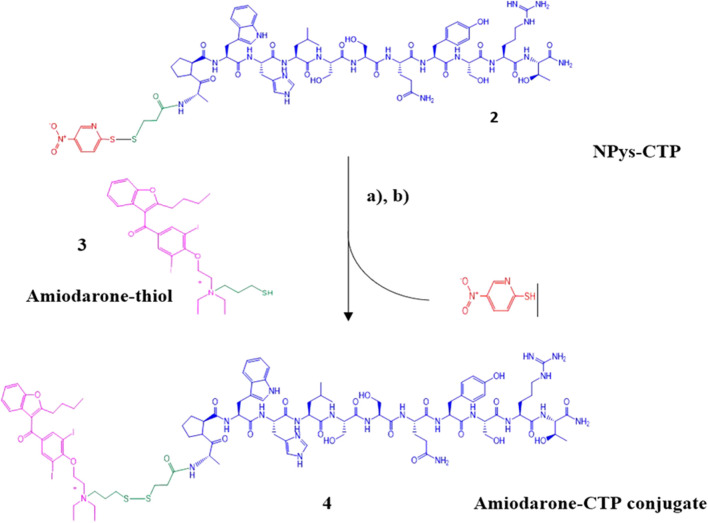
Synthesis of Amiodarone-CTP conjugate. **(A)** Npys-S-CTP, 20% 1 M ammonium acetate in DMF, 2 h at room temperature; **(B)** Preparative HPLC purification followed by MALDI analysis.

Pure Amiodarone-thiol 3 was conjugated to NPys-S-CTP 2 in 20% 1 M ammonium acetate buffer (pH 4.0)/DMF at room temperature for 2 h with gentle mixing (Scheme 4). The crude Amiodarone-CTP conjugate 4 was then purified using preparative C-18 RP-HPLCand the resulting fractions pooled into 50 mL polypropylene conical tubes and shell frozen in dry ice/ethanol followed by overnight lyophilization.

Determination of purity was accomplished using analytical C-18 RP-HPLC (Supplementary Figure S7). Final conjugate yield based on the initial 1 mmol synthesis scale was approximately 180 mg (13.4%) based on the initial purified CTP-thiol intermediate. MALDI analysis was performed to confirm identity of the Amiodarone-CTP conjugate product (Supplementary Figure S8). Despite our best efforts, the various MALDI conditions applied led to fragmentation of the final product with peaks showing at m/z 1725.89 + 519.09, indicating a m/z of 2244 of the final conjugate (2241.04 calculated for [M + H]). A second set of fragmentation products at m/z 646.05 and m/z 1541.85 are presumably the result of fragmentation of the conjugate at both the disulfide linker and quaternary amine resulting in the masses of Amiodarone at m/z 646.05 and CTP-thiol at m/z 1519.85 [M + H] including 1541.85 for [M + Na] observed (Supplementary Figure S8).

### Conjugate stability study

**FIGURE 2:**
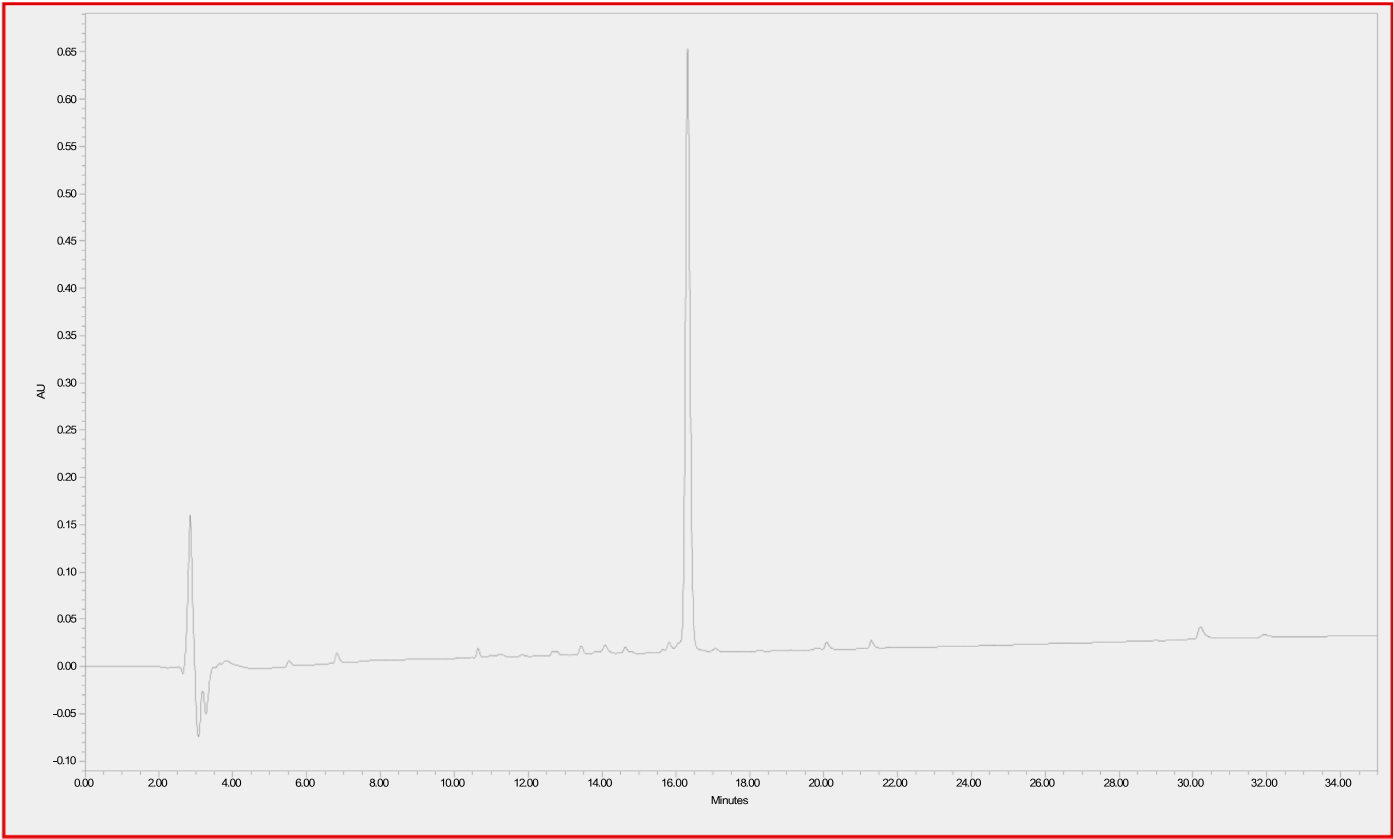
CTP-SS-Amiodarone in perfusion buffer at 37°C at time 0 h.

**FIGURE 3:**
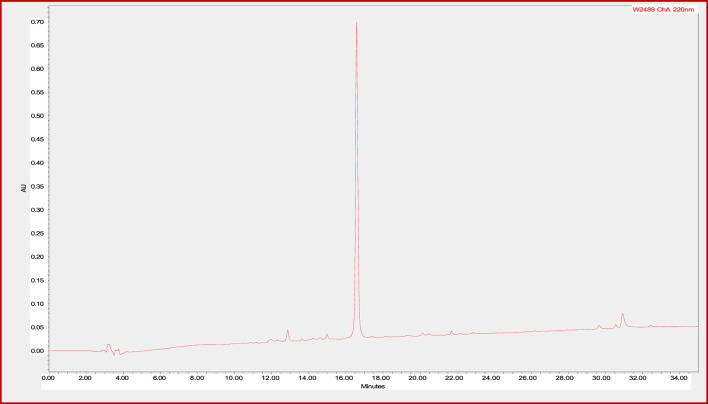
CTP-SS-Amiodarone in perfusion buffer at 37°C for 7 days.

In order to determine the stability of the CTP-amiodarone conjugate 4 to the conditions required for future perfusion studies in animal models, the conjugate was incubated in guinea pig-heart perfusion buffer for 7 days at 37°C. HPLC analysis of the conjugate at baseline or time 0 (Figure 2) and 7-day time points (Figure 3) demonstrated that the disulfide bond linker strategy is stable under these conditions which would be a prerequisite to *in vivo* studies of conjugate efficacy.

### HPLC characterization study

Due to the demonstrated tendency of purified Amiodarone-CTP (Supplementary Figure S9A) to undergo fragmentation under the conditions required for characterization using MALDI mass spectrometry, we devised a simple HPLC method to further characterize the product using retention time comparisons to the purified synthesis intermediates. Cleavage of the disulfide bridge containing linker of Amiodarone-CTP using the reducing agent Dithiothreitol (DTT) would be anticipated to generate the two fragments CTP-thiol and Amiodarone-thiol (Supplementary Figure S9B, C respectively). To demonstrate this assumption, a 1 mg/mL solution of Amiodarone-CTP conjugate was prepared in PBS/50% Trifluoroethanol at pH7.4 containing 10 mM Dithiothreitol (DTT) and allowed to gently mix at room temperature for 4 h. The mixture was then directly injected and analyzed using the analytical HPLC system. The anticipated fragmentation to CTP-thiol and Amiodarone-thiol fragments of the conjugate was demonstrated as depicted in Supplementary Figure S9D.

### *In Vivo* studies

**FIGURE 4:**
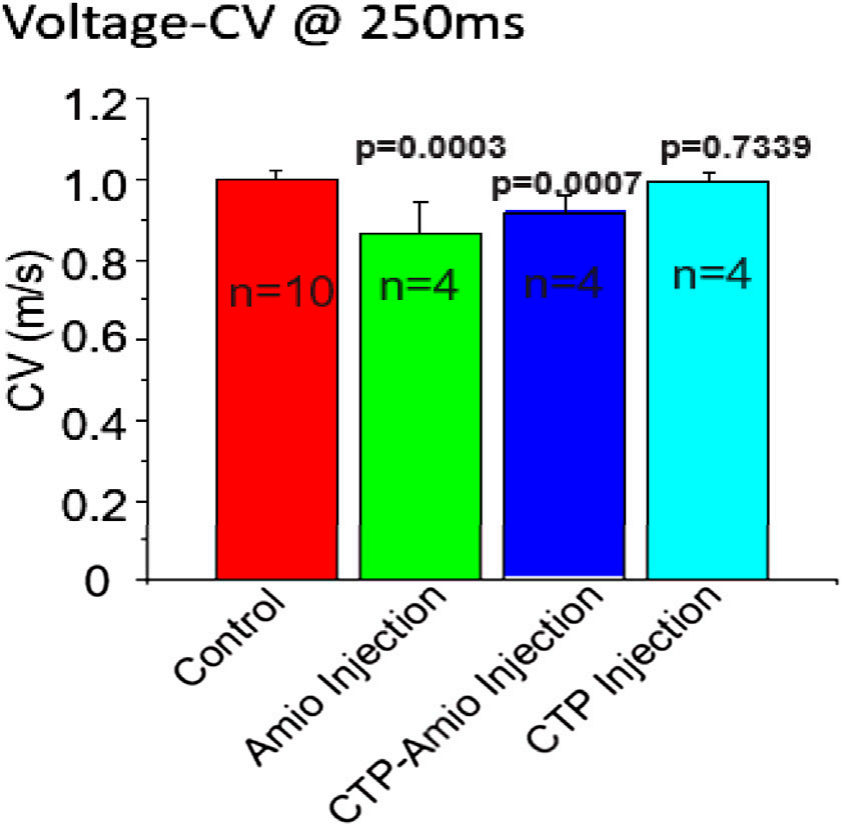
Conduction velocities in guinea pig hearts treated with vehicle only (control; *n* = 10), amiodarone (*n* = 4), CTP-amiodarone (*n* = 4), or CTP alone (*n* = 4). All two-tailed p-values are for pair-wise comparisons to control animals.

All animal protocols were approved by University of Pittsburgh’s institutional animal care and use committee. Adult, male (250-350 gm) guinea pigs were injected daily with either vehicle only, Amiodarone (80 mg/kg) for 7 days, CTP-amiodarone at 1/10th the molar dose for 5 days, or CTP only for 5 days. All injections were intraperitoneal injections. At the end of treatments, guinea pigs were euthanized, hearts dissected, and immediately placed in a Langendorff’s perfusate apparatus where conduction velocities could be measured. As shown in Figure 4, amiodarone, as predicted, decreased conduction velocities significantly as compared to control hearts (*p* = 0.0003), as did CTP-amiodarone significantly (*p* = 0.0007) at 1/15th the total molar dose (amiodarone treatment for 7 days, *versus* 1/10th the molar dose of CTP-amiodarone for 5 days). This phenomenon was not observed with CTP only injections (Figure 4). We interpreted this as CTP-Amiodarone having physiological effects expected of Amiodarone alone but at a much lower dose.

## Discussion

Newly developed targeted strategies are the future for the option of utilizing Amiodarone as a long-term therapeutic due to the significant anti-arrhythmic potential of this drug. As with many new technological advancements there are inevitable limitations to be expected with each new technology. For instance, amiodarone hydrogels require application directly to the epicardium of the heart (Muratovska and Eccles, 2004; Ahmed et al., 2019; Bockeria et al., 2020; Motawea et al., 2021) which may be of great utility in certain circumstances such as during complex surgical procedures, but are not practical for routine, long-term treatment in non-surgical patients. Amiodarone loaded cyclodextrin nanoparticles (Li et al., 2018) efficiently increase cardiac uptake of amiodarone in macrophage abundant tissues such as in the inflamed heart in the setting of myocarditis, resulting in decreased severity of off-target systemic toxicity, but cannot target the cardiomyocytes of the non-inflamed heart, where the resident macrophage population is negligible. In a similar manner, Amiodarone loaded Poly (lactic-co-glycolic acid (PLGA) nanoparticles increase solubility of the lipophilic amiodarone drug resulting in controlled release which reduces systemic toxicity (Pereira et al., 2015) but are also not exclusive to the targeting of cardiac tissues. Peptide-conjugated Amiodarone prodrugs such as Amiodarone-CTP will increase the solubility of Amiodarone while at the same time being biologically compatible while reducing systemic toxicity and targeting cardiomyocytes, the seat of the pathology of rhythm disturbances of the heart. Non-specific CPPs are taken up by myriad different cell types, with some, like Tat (Schwarze et al., 1999), even crossing the blood-brain barrier and would not increase specific uptake by cardiomyocytes. One major obstacle to the use of peptide-based prodrugs is the inherent instability to oral administration. Future constructs could eventually combine the sustained release properties of nanoparticles with the specific targeting potential of Amiodarone-CTP prodrugs, such as encapsulation in PLGA nanoparticles for instance and result in a therapeutic with all the desired properties such as oral administration, or depot subcutaneous injections leading to high specificity along with efficacious delivery to treat arrhythmias. The successful production, testing, upscaling of CTP-Amiodarone has the potential for being the first targeted therapy for arrhythmias leading to need for a substantially smaller dose, and less off-target toxicities due to a lower dose as well as less uptake by organs other than the heart. As a proof of principle, this can lead to further advances in next-generation therapeutic strategies for the maintenance of cardiac rhythm and other cardiac pathologies. Our data presented here shows the feasibility of this approach, our successful conjugation of amiodarone to the N-terminus of CTP, with *in vivo* data suggesting delivery of amiodarone to the heart with resulting changes expected of amiodarone observed, though at ∼1/15th the total molar dose of amiodarone. We are now poised to test this conjugate in an animal model of a relevant cardiac arrhythmia, such as atrial fibrillation, for treatment efficacy.

The ability to incorporate different linker systems with a variety of physical properties such as chain length hydrophilicity/hydrophobic character will only improve the selectivity of this conjugate towards cardiomyocytes. As an example, grafting polyethylene glycol (PEG) based thiol containing linkers into the amiodarone pro-drug constructs may lead to future conjugates with enhanced bioavailability to the heart with minimal systemic off-target drug interactions. Incorporation of various dyes or radioisotope labels into the Amiodarone-prodrug structure could allow for the elucidation of biodistribution and confirm targeted delivery *in vivo* utilizing single-photon emission computerized tomography or positron emission nuclear imaging. Confirmation of target engagement in rodent models will precede the testing in human subjects as the technology is advanced from bench to bedside.

## Methods and materials

**SCHEME 5:**
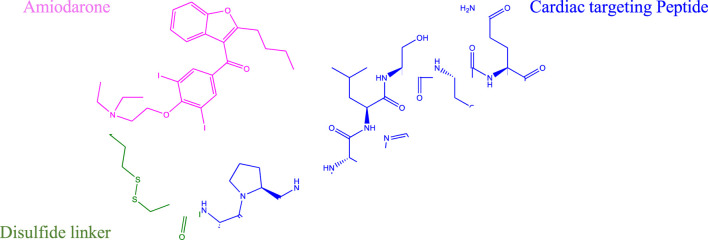
Schematic representation of the final Amiodarone-SS-CTP conjugate.

All data generated or analyzed during this study are included in this published article and its Supporting Material files. The conversion of tertiary amine containing compounds to quaternary ammonium salts by reaction with alkyl halides is known as the Menschutkin reaction. This reaction leads to very stable compounds with specialized properties important to materials science and numerous other applications (Okeke et al., 2019). Kris et al. applied the Menschutkin reaction to the formation of N-Phosphonooxymethyl functionalized quaternary amine prodrugs from tertiary amine containing drugs such as Amiodarone using tert-butyl chloromethyl phosphate intermediates (Krise et al., 1999). Building on this technology we synthesized a thiolated Amiodarone intermediate via the covalent attachment of a chloroalkylthiol to Amiodarone hydrochloride. The resultant Amiodarone-thiol was subsequently linked to a thiol-modified intermediate of the well-established cardiac targeting peptide (CTP) (14) through a disulfide linker to form the CTP-amiodarone prodrug (Scheme 5).

### Chemicals

All solvents and reagents used were purchased from commercial sources and used without further purification. Fmoc-Ala-OH, Fmoc-Pro-OH, Fmoc-Trp (Boc)-OH, Fmoc-His (Trt)-OH, Fmoc-Leu- OH, Fmoc-Ser(tBu)-OH, Fmoc-Gln (Trt)-OH, Fmoc-Tyr (tbu)-OH, Fmoc-Arg (Pbf)-OH, Fmoc- Thr (tBu)-OH and Oxyma-Pure were ordered from Novabiochem (Millipore Sigma Corporation2,2,2-Trifluoroethanol (TFE), Dichloromethane (DCM), Dimethylformamide (DMF), Acetonitrile and Diethyl Ether (Et2O) were ordered from Fisher Scientific (Hampton, NH, United States). Trifluoroacetic acid (TFA), Diisopropylethylamine (DIPEA) and Piperidine were ordered from Chem-Impex International (Wood Dale, IL, United States). Amiodarone HCL, 3-(Tritylthio) propionic acid, Ethanedithiol (EDT), Triisopropylsilane (TIPS), 3-chloropropanethiol, 1,2,2,6,6- Pentamethylpiperidine and Sodium Iodide were ordered from Sigma-Aldrich (St. Louis, MO, United States). 2-(1H-Benzotriazole-1-yl)-1,1,3,3-tetramethylaminium tetrafluoroborate (TBTU) and 1- Hydroxybenzotriazole hydrate (HOBt) were ordered from P3 Biosystems (Louisville, KY, United States)., St. Louis, MO). Rink amide MBHA resin was ordered from vivitide, LLC (Garder, MA, United States).

### Equipment

All purification steps were performed on a Waters Delta Prep 4,000 chromatography system equipped with a Phenomonex Gemini-NX C-18 21.2 × 250 mm column. Analytical characterization of peptide intermediates and conjugate stability testing was performed on a Waters Alliance chromatography system using a Waters Xbridge BEH protein 4.5 × 250 mm C4 columm. A Bruker Ultra FlexTreme Tof/Tof MS and workstation was utilized for MALDI confirmation of expected mass for all synthetic intermediates. Lyophilization steps were performed on an SP Virtis BenchTop Pro with Omnitronics Freeze dryer unit. Centrifugation steps were accomplished using a Thermoscientific benchtop microcentrifuge unit.

### Analytical RP HPLC

Preparation of samples for analytical evaluation of purity at the various synthesis intermediate steps involved dissolving each sample at a concentration of 1 mg/ml in 50%TFE/0.1%TFA and performing the separation on a Waters Alliance chromatography system using standard Acetonitrile-0.1% TFA gradient conditions at a flow rate of 1 mL/min.

### Preparative RP HPLC

Purification of synthetic intermediates and the final Amiodarone-SS-CTP conjugate was performed by dissolving 20 mg batches in 50%TFE/0.1%TFA at a concentration of 1 mg/ml. These large-scale separations were accomplished on a Waters Delta Prep 4,000 chromatography system using standard Acetonitrile-0.1% TFA gradient conditions at a flow rate of 25 mL/min.

### MALDI analysis

Sample preparation for characterization by MALDI involved dissolution of each sample of interest in 50%ACN/0.1% TFA at a concentration of 1 mg/ml. 2,5-Dihydroxy Benzoic acid (DHB) matrix was then prepared at a concentration of 10 mg/ml in 50%ACN/0.1%TFA. Samples were then diluted 1:1 with DHB matrix solution and spotted onto a MALDI target plate followed by measurement of m/z on a Bruker Ultra FlexTreme Tof/Tof MS.

### *In Vivo* studies

All animal studies were approved by the University of Pittsburgh’s institutional animal care and use committee. All methods were carried out in accordance with relevant institutional animal care and use guidelines and regulations. We complied with all the ARRIVE guidelines. Adult, male, 6-week-old guinea pigs (250-350 gm body weight) were acclimatized for 48-h prior to initiating experiments. Animals were injected with 80 mg/kg of amiodarone daily intra-peritoneally for 7 days, a dosing regimen shown in animal studies to induce conduction velocity changes (Sosunov et al., 1996; Garcia et al., 2018; Bosch et al., 1999), or 1/10th the molar dose of CTP-amiodarone for 5 days, or 1/10th the molar dose of CTP for 5 days. Vehicle treated animals were used as controls. At the end of the treatment period, animals were euthanized, hearts excised and perfused in a Langendorff apparatus with Tyrode’s solution containing (in mM): NaCl (130), KCl (4), MgSO4 (1.2), NaHCO3 (25), Glucose (5), CaCl2 (1.25), Mannitol (45) gassed with 95% O2 and 5% CO2, pH 7.0 at 37°C. Hearts were placed in a custom-designed chamber to subdue motion artifacts, along with a 15 min blebbistatin (5 µM) in perfusate to minimize contractions. Bolus injections of voltage (RH237, 25 µL of 2 mg/mL dimethyl sulfoxide (DMSO)) and Ca2+-indicator dye (Rhod-2/AM, 200 µL of 2 mg/mL DMSO) were injected in the air-trap above the aortic cannula. Fluorescence from the epicardium was collected with a camera lens, split with a 570 nm dichroic mirror and focused on two CMOS cameras (Sci-Media UltimaOne) capturing at the fluorescence emission at 570–595 nm for cytosolic Ca2+ and 610–750 nm wavelengths for voltage. After resting sinus rhythm image acquisition, hearts were paced at 250 m with programmed stimulation to measure and compare conduction velocity and transient duration (90%).

## Conclusion

We present here a method for conjugating amiodarone, a common anti-arrhythmic, to a cardiomyocyte targeting cell penetrating peptide via a disulfide bond, to demonstrate the feasibility of producing targeted therapeutics with a prodrug approach. The disulfide bond, being a covalent one, is stable at 37°C for 7 days, and likely beyond. Our rationale is to use the peptide as a vector to deliver our therapeutic of interest, Amiodarone, to cardiomyocytes, where upon internalization the disulfide bond is broken in the reducing intracellular environment, releasing the drug. Such an approach would lead to targeted therapy of arrhythmias with a lesser dose requirement, and lesser off-target effects associated with long-term amiodarone use. Based on our results, testing CTP- amiodarone conjugate in a relevant animal model of a common cardiac arrhythmia like atrial fibrillation is indicated.

## Data Availability

The original contributions presented in the study are included in the article/Supplementary Material, further inquiries can be directed to the corresponding author.
