# Supplementary material for: Conjugation of amiodarone to a novel cardiomyocyte cell penetrating peptide for potential targeted delivery to the heart
Source: Front Chem. 2023 Jul 12;11:1220573. doi: 10.3389/fchem.2023.1220573 (PMC10402922; doi:10.3389/fchem.2023.1220573)
Supplement: Supplementary file 1 [file DataSheet1.docx]

**Supplementary Material**

10.451

11.503

12.583

AU

0.00

0.20

0.40

0.60

0.80

1.00

1.20

1.40

1.60

1.80

2.00

2.20

2.40

2.60

2.80

3.00

Minutes

0.00

2.00

4.00

6.00

8.00

10.00

12.00

14.00

16.00

18.00

20.00

22.00

24.00

26.00

28.00

30.00

32.00

34.00

**Figure S1: HPLC Chromatogram of CTP-thiol**


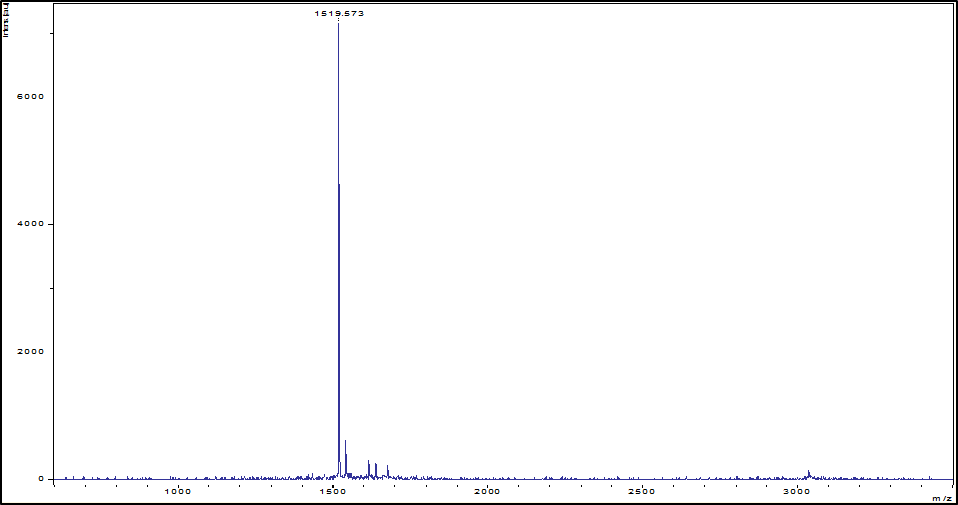

**Figure S2: MALDI analysis of CTP-thiol**

13.444

13.867

14.516

AU

-0.20

0.00

0.20

0.40

0.60

0.80

1.00

1.20

1.40

1.60

1.80

2.00

2.20

2.40

2.60

2.80

3.00

3.20

3.40

Minutes

0.00

2.00

4.00

6.00

8.00

10.00

12.00

14.00

16.00

18.00

20.00

22.00

24.00

26.00

28.00

30.00

32.00

34.00

**Figure S3: HPLC Chromatogram of NPys-S-CTP**

#

#

#

#

# Figure S4: MALDI analysis of NPys-S-CTP

19.609

Absorbance (220 nm)

-0.20

0.00

0.20

0.40

0.60

0.80

1.00

1.20

1.40

1.60

1.80

2.00

2.20

2.40

2.60

2.80

3.00

3.20

3.40

3.60

Time (Minutes)

0.00

1.00

2.00

3.00

4.00

5.00

6.00

7.00

8.00

9.00

10.00

11.00

12.00

13.00

14.00

15.00

16.00

17.00

18.00

19.00

20.00

21.00

22.00

23.00

24.00

25.00

26.00

27.00

28.00

29.00

30.00

31.00

32.00

33.00

34.00

35.00

# Figure S5: HPLC Chromatogram of Amiodarone-thiol

#

# Figure S6: MALDI analysis of Amiodarone thiol

16.040

AU

0.00

0.20

0.40

0.60

0.80

1.00

1.20

1.40

1.60

1.80

2.00

2.20

2.40

2.60

2.80

3.00

3.20

Minutes

0.00

5.00

10.00

15.00

20.00

25.00

30.00

35.00

**Figure S7: Amiodarone-SS-CTP-amide HPLC Trace**

**
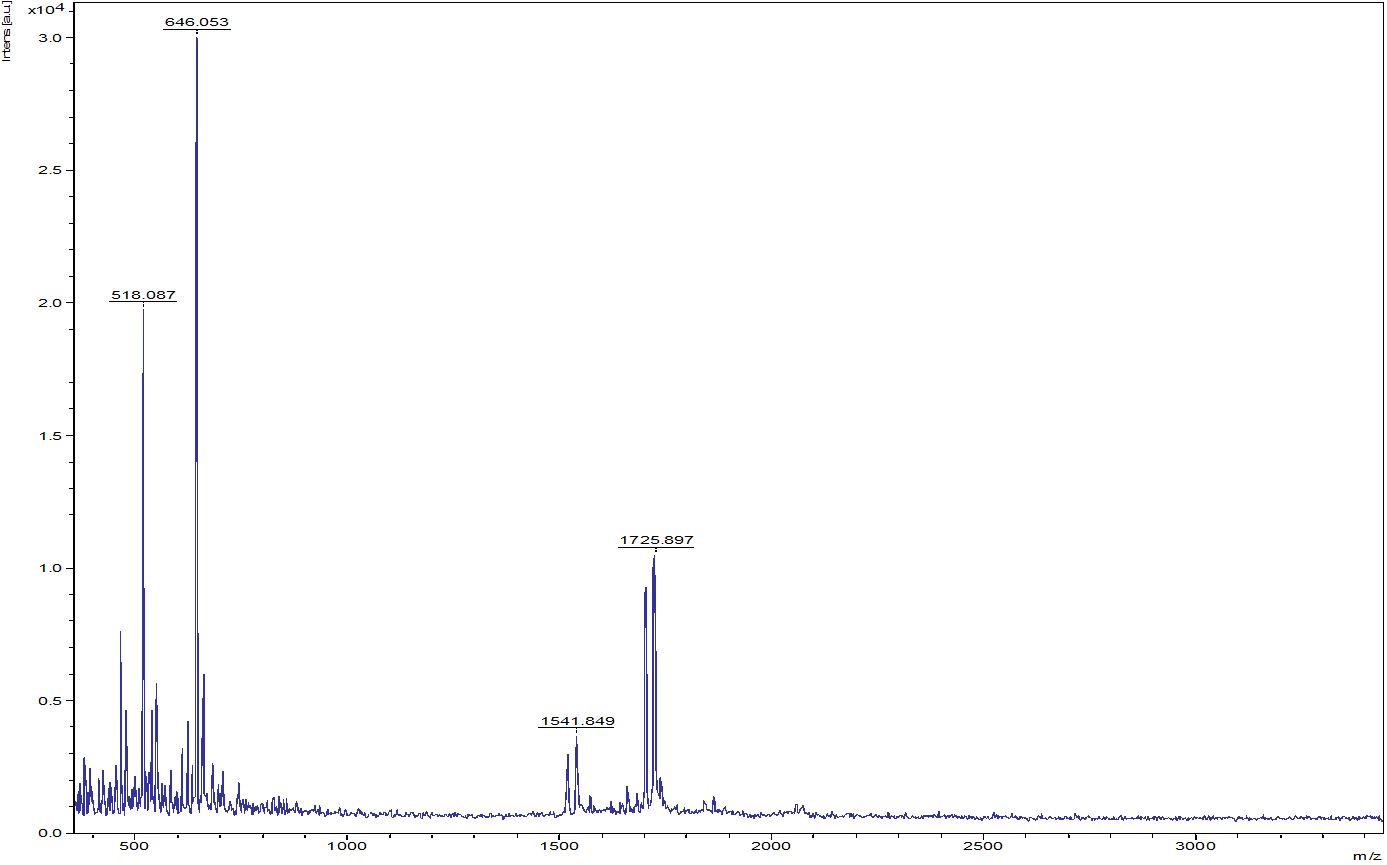
**

**Figure S8: Amiodarone-SS-CTP-MALDI**

**HPLC characterization study after deliberate cleavage with Dithiothreitol (DTT) (Figures S9a-S9d)**

16.040

AU

0.00

0.20

0.40

0.60

0.80

1.00

1.20

1.40

1.60

1.80

2.00

2.20

2.40

2.60

2.80

3.00

3.20

Minutes

0.00

5.00

10.00

15.00

20.00

25.00

30.00

35.00

**Amiodarone-CTP**

**Figure S9-a: Amiodarone-CTP purified**

10.451

11.503

12.583

AU

0.00

0.20

0.40

0.60

0.80

1.00

1.20

1.40

1.60

1.80

2.00

2.20

2.40

2.60

2.80

3.00

Minutes

0.00

2.00

4.00

6.00

8.00

10.00

12.00

14.00

16.00

18.00

20.00

22.00

24.00

26.00

28.00

30.00

32.00

34.00

**CTP-Thiol**

**Figure S9-b: CTP-Thiol purified**

19.609

Absorbance (220 nm)

-0.20

0.00

0.20

0.40

0.60

0.80

1.00

1.20

1.40

1.60

1.80

2.00

2.20

2.40

2.60

2.80

3.00

3.20

3.40

3.60

Time (Minutes)

0.00

1.00

2.00

3.00

4.00

5.00

6.00

7.00

8.00

9.00

10.00

11.00

12.00

13.00

14.00

15.00

16.00

17.00

18.00

19.00

20.00

21.00

22.00

23.00

24.00

25.00

26.00

27.00

28.00

29.00

30.00

31.00

32.00

33.00

34.00

35.00

**Amiodarone-thiol**

**Figure S9-c: Amiodarone-thiol purified**


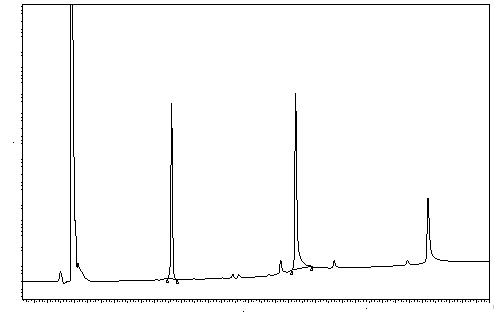


**CTP-Thiol**

**Amiodarone-Thiol**

**Figure S9-d: Amiodarone-CTP cleavage products after DTT reduction**
